# Ising-like models for stacking faults in a free electron metal

**DOI:** 10.1098/rspa.2020.0319

**Published:** 2020-10-28

**Authors:** Martina Ruffino, Guy C. G. Skinner, Eleftherios I. Andritsos, Anthony T. Paxton

**Affiliations:** Department of Physics, King’s College London. Strand, London WC2R 2LS, UK

**Keywords:** magnesium, total energy, convergence, precision, electronic structure, stacking faults

## Abstract

We propose an extension of the axial next nearest neighbour Ising (ANNNI) model to a general number of interactions between spins. We apply this to the calculation of stacking fault energies in magnesium—particularly challenging due to the long-ranged screening of the pseudopotential by the free electron gas. We employ both density functional theory (DFT) using highest possible precision, and generalized pseudopotential theory (GPT) in the form of an analytic, long ranged, oscillating pair potential. At the level of first neighbours, the Ising model is reasonably accurate, but higher order terms are required. In fact, our ‘ AN^*N*^NI model’ is slow to converge—an inevitable feature of the free electron-like electronic structure. In consequence, the convergence and internal consistency of the AN^*N*^NI model is problematic within the most precise implementation of DFT. The GPT shows the convergence and internal consistency of the DFT bandstructure approach with electron temperature, but does not lead to loss of precision. The GPT is as accurate as a full implementation of DFT but carries the additional benefit that damping of the oscillations in the AN^*N*^NI model parameters are achieved without entailing error in stacking fault energies. We trace this to the logarithmic singularity of the Lindhard function.

## 1. Introduction

It is a 100 years since Wilhelm Lenz suggested a problem in magnetism to his student Ernst Ising, resulting in the now famous eponymous model [1] which has become a paradigm in statistical mechanics. Over 30 years ago, the Ising model and its extension to second neighbours, the axial next nearest neighbour Ising (ANNNI) model [2], found an application in the understanding of planar faults in semiconductors [3] and later in metals [4]. Moreover, in the study of semiconductors, the model was extended to third neighbours [5,6]. In addition to stacking fault energies (SFEs), the theory has been applied to antiphase boundaries [7,8]. In the simplest case of the comparison in stability of face centred cubic (fcc) versus hexagonal close-packed (hcp) lattices, fcc corresponds to the ‘ferromagnetic’ arrangement of spins, ·· · ↑ · · ·, and is stable in the Ising model for *J* > 0; and hcp is represented by ‘antiferromagnetic’ spin arrangement, ·· · ↑ ↓ · · ·, and is stable if *J* < 0. If the parameters of the ANNNI model differ in sign then the opportunity for competing interactions arises resulting in complexity in the phase diagram [2] and this has been suggested as an origin of the many high temperature modulated phases in binary alloys [9], first discovered in the same year as the Ising model by Johannson & Linde [10].

In this work, we study Ising-like models applied to the free electron metal magnesium. Planar faults in Mg are of great interest in problems of plasticity mediated by slip and twinning, effects of temperature and the role of alloying elements. It is curious that Mg alloys, having the lowest density among structural metals, are not as widely used as their low cost and high strength to weight ratio would suggest. One reason for this is difficulty in forging, as there is only a narrow temperature range in which this is possible; and their tendency during metallurgical processing to develop deleterious crystallographic textures. Present-day alloy design is targeted at formable, low cost alloys. From the viewpoint of fundamental theory, what singles out Mg in the present context is its electronic structure being free electron-like. This has the benefit that an analytic pair potential exists that emerges out of the density functional theory (DFT) [11,12]. On the other hand, this pair potential is long ranged as is to be expected from the logarithmic singularity in the Lindhard response function [13]. As a consequence, the natural question arises as to the validity of Ising and ANNNI models which are truncated after one or two neighbours. Our approach here is to propose an ‘ AN^*N*^NI model’, which is an extension of the ANNNI model to interactions out to *N* neighbours. We develop this model with formulae for the four principal basal faults in hcp metals, up to *N* = 6. By making the most precise estimates of the parameters of the AN^*N*^NI model using electronic structure theory, we are able to assess the precision and the convergence of the Ising-like expansion.

The structure of this paper is as follows. We describe the basal planar faults peculiar to hcp metals in §2. Theory is presented in §3, with §3a on the Ising-like models; §3b on the total energy methods which we employ in this work. We present our results in §4, and Discussion and Conclusions will be found in §§5 and 6.

## 2. Planar faults in hcp metals

In hcp magnesium, there are four stable stacking faults in the basal plane, as described in Hirth & Lothe [14]. The first intrinsic stacking fault I_1_ (or growth fault), is obtained through the removal of a basal plane, followed by the shear of the crystal above the fault of $\frac{1}{3}a$ in the $\left[\bar{1}100\right]$ direction, where *a* is the hcp basal lattice constant (nearest neighbour distance). The second intrinsic stacking fault I_2_ (or deformation fault) is formed by a shear of $\frac{1}{3}a$ in the $\left[\bar{1}100\right]$ direction; it is this fault which separates partial dislocations of the 〈*a*〉 dislocation dissociated on the basal plane. The extrinsic stacking fault, E, is obtained by inserting an extra plane in the perfect sequence; the twin-like fault, T, possesses mirror symmetry about the fault plane [15] and is formed by replacing an A plane with a C plane [16] (see §3a). Experimental measurements of stacking fault energies are notoriously difficult and in Mg only upper bounds have been established. Hence, there has been a heavy reliance on first principles calculations based on DFT [17], with the main approach being the ‘supercell’ method [15,18] (§3biii).

## 3. Theory

### (a) Ising-like models

Many close-packed metals and alloys and many semiconductors based in the zincblende or wurtzite prototypes have crystal structures that correspond to the stacking of planes of close-packed spheres. Such hexagonal, or honeycomb, planes possess hollow sites labelled ‘A’ and ‘B’ which may receive a sphere from a subsequent plane, stacked atop the first, in such a way as to maintain close packing. If the receiving plane is designated ‘C’ then a close-packed repeating unit cell may be represented by a sequence of A, B and C designations which uniquely specify the crystal [19]. For example, ·· · ABC · · · corresponds to the stacking of an fcc metal, while ·· · AB · · · describes the ideal hcp crystal. In the case of the diamond lattice, the stacking is that in fcc, on the understanding that each close-packed ‘sphere’ is actually an atom plus a further basis atom. The scheme is applicable also to ordered alloys based on the fcc lattice and not necessarily restricted to the stacking of close-packed planes—for example, the *L*1_2_ *D*0_22_ and *L*1_0_ superlattices [4,7,8].

If a crystal is constructed from a stacking sequence of atomic planes then its total energy may be written as the sum of the interaction energies between planes plus a contribution coming from the energy of the system when interactions are set to zero. A spin *S* = ±1 is assigned to the *i*th plane, according to whether the plane *i*+1 follows the ‘correct’ stacking sequence or not. Conventionally, we assert that the correct stacking sequence is A → B → C → A, whereas the incorrect one is A → C → B → A. Consequently, the energy of a crystal with an arbitrary stacking sequence is expressed as follows:

3.1 $$E=E_{0}-\sum_{n}^{N}J_{n}\sum_{i}^{M}S_{i}S_{i+n},$$

with *E*_0_ being the energy of the system when all interactions between planes are set to zero, *M* being the number of planes and *N* being the furthest neighbour interaction to be taken into account. The *J*_*n*_ parameters are the interaction coefficients between neighbouring layers, such that e.g. *J*_1_ represents the interaction energy between two layers which are nearest neighbours. Here, we assume that the magnitude of the interaction energy between neighbours decreases as *n* increases. Truncated at the first term in the sum over *n*, equation (3.1) is the well-known Ising model [1]. In the ANNNI model [2], interactions up to *J*_2_ are taken into account. In this work, we propose to observe the behaviour of the *J* parameters as further neighbours are added. We call these A${\text{N}}^{N}$NI models, with *N* = 1, 2, 3, 4, 5, 6 depending on how many neighbours are included. The sums in equation (3.1) can be evaluated for perfect structures straightforwardly; we have chosen seven structures, classified in terms of the type of unit cell as in the case of the polytypes of silicon carbide [5]. We label each structure with a number, representing the number of planes in the unit cell, followed by a letter, representing the Bravais lattice type, as in the Ramsdell classification [20]. The chosen polytypes and their stacking sequences are listed in table 1. It should be noted that the 2H structure is identical to hcp; while the 3R structure is identical to fcc, although it is no longer cubic if the material has non-ideal *c*/*a* ratio. In fact, the AN^*N*^NI model applied to an hcp metal need not employ the ideal axial ratio of $c/a=\sqrt{8/3}$, as long as the interplanar spacing of all the polytypes is adjusted to match that of the hcp, antiferromagnetic, ground state. In the present work, we use the axial ratio that is predicted by the electronic structure theory, see §§3bi and ii.

**Table 1.** Stacking sequences and ‘spin’ arrangements of the seven polytypes of magnesium used in the AN^*N*^NI model, as well as the disruptions to the 2H sequence that define the four types of stacking faults. I_1_ and I_2_ are intrinsic and E is the extrinsic fault; T is the twin. 4H is also known as ‘double hcp’.

structure	stacking sequence	spin arrangement
2H	AB	·· · ↑ ↓ · · ·
3R	ABC	·· · ↑ · · ·
4H	ABCB	·· · ↑ ↑ ↓ · · ·
6H	ABCACB	·· · ↑ ↑ ↑ ↓ · · ·
9R	ABCBCACAB	·· · ↑ ↑ ↓ ↑ ↑ ↓ ↑ ↑ · · ·
10H	ABCABCBACB	·· · ↑ ↑ ↑ ↑ ↑ ↓ ↓ ↓ ↓ · · ·
15R	ABCACBCABACABCB	·· · ↑ ↑ ↑ ↓ ↓ ↑ ↑ ↑ ↓ ↓ ↑ ↑ ↑ ↓ · · ·
I_1_ fault	·· · ABABCBCB · · ·	·· · ↑ ↓ ↑ ↓ ↑ ↑ ↓ ↑ ↓ ↑ ↓ · · ·
I_2_ fault	·· · ABABCACA · · ·	·· · ↑ ↓ ↑ ↓ ↑ ↑ ↑ ↓ ↑ ↓ ↑ · · ·
E fault	·· · ABABCABAB · · ·	·· · ↑ ↓ ↑ ↓ ↑ ↑ ↑ ↑ ↓ ↑ ↓ ↑ · · ·
T fault	·· · ABABCBABA · · ·	·· · ↑ ↓ ↑ ↓ ↑ ↑ ↓ ↓ ↑ ↓ ↑ ↓ · · ·

Evaluating the sums in equation (3.1) up to *N* = 6 and dividing by the number of planes *M*, we obtain the energies per plane of each polytype

3.2 $$\begin{array}{r} E_{2\text{H}}=J_{0}+J_{1}-J_{2}+J_{3}-J_{4}+J_{5}-J_{6}, \end{array}$$

3.3 $$\begin{array}{r} E_{3\text{R}}=J_{0}-J_{1}-J_{2}-J_{3}-J_{4}-J_{5}-J_{6}, \end{array}$$

3.4 $$\begin{array}{r} E_{4\text{H}}=J_{0}+J_{2}-J_{4}+J_{6}, \end{array}$$

3.5 $$\begin{array}{r} E_{6\text{H}}=J_{0}-\frac{1}{3}J_{1}+\frac{1}{3}J_{2}+J_{3}+\frac{1}{3}J_{4}-\frac{1}{3}J_{5}-J_{6}, \end{array}$$

3.6 $$\begin{array}{r} E_{9\text{R}}=J_{0}+\frac{1}{3}J_{1}+\frac{1}{3}J_{2}-J_{3}+\frac{1}{3}J_{4}+\frac{1}{3}J_{5}-J_{6}, \end{array}$$

3.7 $$\begin{array}{r} E_{10\text{H}}=J_{0}-\frac{3}{5}J_{1}-\frac{1}{5}J_{2}+\frac{1}{5}J_{3}+\frac{3}{5}J_{4}+J_{5}+\frac{3}{5}J_{6}, \end{array}$$

3.8 $$\begin{array}{r} \;\text{and}\;E_{15\text{R}}=J_{0}-\frac{1}{5}J_{1}+\frac{3}{5}J_{2}+\frac{3}{5}J_{3}-\frac{1}{5}J_{4}-J_{5}-\frac{1}{5}J_{6}, \end{array}$$

where *J*_0_ = *E*_0_/*M* is the energy of one plane if interactions are not taken into account.

The same can be done for crystals containing stacking faults by describing them as limiting structures with periodic behaviour [3,5,21]; hence, each type of stacking fault is represented as a disruption in the 2H sequence, as listed in table 1. For example, evaluating the sum in equation (3.1) for the I_1_ stacking fault and dividing by *M*, we obtain

3.9 $$E_{{\text{I}}_{1}}(M)=J_{0}+\frac{M-2}{M}J_{1}-\frac{M-4}{M}J_{2}+\frac{M-6}{M}J_{3}-\frac{M-8}{M}J_{4}+\frac{M-10}{M}J_{5}-\frac{M-12}{M}J_{6}.$$

The stacking fault energies are then defined by Denteneer & van Haeringen [3] as the excess energy compared to the perfect crystal

3.10 $$\Delta E_{\text{SFE}}=\lim_{M\to \infty }2M[E_{\text{SFE}}(2M)-E_{\text{2H}}],$$

where the factors of 2 account for the periodicity of the crystal.

Applying this to equation (3.9) and similarly for the other faults, we obtain

3.11 $$\begin{array}{r} \Delta E_{{\text{I}}_{1}}=-2J_{1}+4J_{2}-6J_{3}+8J_{4}-10J_{5}+12J_{6}, \end{array}$$

3.12 $$\begin{array}{r} \Delta E_{{\text{I}}_{2}}=-4J_{1}+4J_{2}-4J_{3}+4J_{4}-4J_{5}+4J_{6}, \end{array}$$

3.13 $$\begin{array}{r} \Delta E_{\text{E}}=-6J_{1}+4J_{2}-6J_{3}+8J_{4}-10J_{5}+12J_{6}, \end{array}$$

3.14 $$\begin{array}{r} \;\text{and}\;\Delta E_{\text{T}}=-4J_{1}+8J_{2}-8J_{3}+8J_{4}-8J_{5}+8J_{6}, \end{array}$$

in agreement with the coefficients found by Wright [6] up to *J*_3_, and here extended to include contributions up to *J*_6_. The *J*-parameters can be expressed in terms of the energies of the perfect structures, and the SFEs can be calculated indirectly, without having to compute the energies of the structures containing the stacking faults, and thus eliminating one of the drawbacks of the supercell model. The SFE is then given as energy per unit area [3], such that *γ* = Δ*E*/*A* with *A* being the area that defines the unit cell in one plane, $A=\left(\sqrt{3}/2\right)a^{2}$. SFEs calculated using this model are independent of cell size: each of the energies of the perfect crystals are calculated from the unit cell of each polytype, and the SFEs are then indirectly calculated as energies per plane, per atom. The cell size-dependency of the supercell method is thus avoided.

### (b) Total energy

A key feature of the AN^*N*^NI model is that a large amount of information is extracted from a small number of total energy calculations on small unit cells. Specifically, calculations of the total energy of *N* + 1 polytypes are needed. Our data are displayed in table 2. It is vital that the greatest possible precision is used. In this work, we have used the DFT. We take two approaches to this. First, we use a standard procedure for obtaining solutions of the Kohn–Sham equations of DFT, here in a basis of linear muffin tin orbitals in a full potential implementation [22]; second, we exploit the fact that Mg is a free electron metal to apply the first principles pair potential formulation of the DFT total energy within the generalized pseudopotential theory (GPT) [11,12,23,24].

**Table 2.** The energies of polytypes of pure Mg, calculated using the GPT; and LMTO-DFT with modified tetrahedron integration [25] and Fermi function smearing with *kT* = 0.03 Ry. The energy of the 2H structure is the binding energy relative to a free atom and is given in eV/atom, and the energies of the other structures are relative to that of 2H, i.e. given as differences in energy also in eV/atom. Note that the use of a large electron temperature leads to an absolute error in total energy of 0.12 eV/atom, while this systematic error clearly cancels in the energy differences between polytypes.

E (eV/atom)	2H	3R	4H	6H	9R	10H	15R
GPT	−1.5926	+0.0107	+0.0054	+0.0070	+0.0031	+0.0084	+0.0063
DFT: tetrahedron	−1.3729	+0.0131	+0.0060	+0.0087	+0.0029	+0.0092	+0.0073
DFT: *kT* = 0.03 Ry	−1.2540	+0.0135	+0.0067	+0.0088	+0.0036	+0.0106	+0.0077

#### (i) LMTO-DFT

We use the full potential linear muffin tin orbital (LMTO) method of Methfessel and van Schilfgaarde [22] as implemented in the questaal code [26,27]. Kohn–Sham orbitals are expanded in a basis set of ‘smooth Hankel functions’ which are atom centred Hankel functions convoluted with Gaussians within a smoothing radius, *R*_sm_. The charge density is expanded in spherical and plane waves. For Mg, we use atomic spheres of radius 3 bohr, and a double energy basis set having Hankel energies of –0.1Ry in *s*, *p* and *d* channels and –0.9Ry in *s* and *p* channels. The smoothing radius is *R*_sm_ = 2 bohr. The plane wave expansion cut-off is 8.2 Ry. Exchange and correlation are calculated using the GGA-PBE [28]. Brillouin zone integration is made using the modified tetrahedron method of Blöchl *et al.* [25]. To achieve the greatest precision, we are absolutely converged with respect to **k**-point sampling: typically dividing the Brillouin zone into 30 divisions along a reciprocal lattice vector of 2*π*/*a*. As an alternative, for reasons given below, for some calculations, we replace tetrahedron Brillouin zone integration with an effective electron temperature of *kT* = 0.03 Ry which acts to smear out the Fermi surface. The LMTO-DFT using tetrahedron integration predicts an equilibrium atomic volume, *Ω*_0_ = 155.3 bohr^3^ and an axial ratio of *c*/*a* = 1.627 and we use these in all LMTO calculations to avoid spurious stresses, especially in the supercells. The lattice constants are in good agreement with the measured low-temperature values, *Ω*_0_ = 153.4 bohr^3^ and *c*/*a* = 1.624 [29]. The axial ratio of Mg is close to the ideal $c/a=\sqrt{8/3}=1.633$.

#### (ii) Generalized pseudopotential theory

The GPT is also a DFT, but here the total energy is cast into a many-atom expansion comprising one, two and further body terms. The expansion is well known to be converged at the two-body term in the free electron metals [23,30]. This means that we can write the total energy as

**Figure 1.**
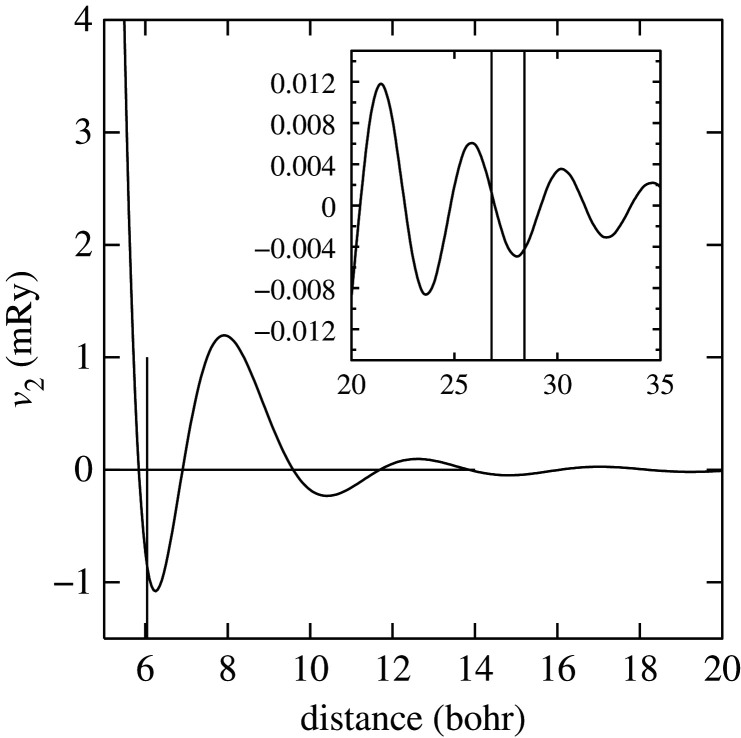
The Mg–Mg pair potential, *v*_2_. A vertical line at *a* = 6.1 bohr indicates the nearest neighbour distance in Mg. The inset shows an expanded scale for the tail of the pair potential; the two vertical lines indicate the start and finish of the cut-off function which damps the pair potential smoothly to zero.

3.15 $$E_{\mathrm{tot}}(\{R\},\Omega )=NE_{\mathrm{vol}}(\Omega )+\frac{1}{2}{\sum_{ij}}^{\prime }v_{2}(R_{ij},\Omega ),$$

which is a function of the average atomic volume *Ω* and the bond lengths, *R*_*ij*_ connecting atoms *i* and *j*. The pair potential *v*_2_ is a function of *Ω*. We should note that since all our calculations in the present work are made at a fixed atomic volume, only the second term in the total energy (3.15) is involved in energy differences; as our structures are all closed packed there is no need for local volume corrections [24]. Detailed expressions for *E*_vol_ and *v*_2_ are given in [12]: *v*_2_(*r*) is proportional to

$$\frac{1}{r}\left(1-\frac{2}{\pi }\int F(q,\Omega )\frac{\sin qr}{r}\;\text{d}q\right),$$

where *F* is the energy–wavenumber characteristic [30,31]. Its oscillations in real space are evident from figure 1: the pair potential is long ranged and oscillating—a typical feature of a metal having a sharp discontinuity in the electron occupation number across the Fermi surface. The oscillations are asymptotically identical to those expected from the Lindhard screening function [13]. Exchange and correlation are treated in the screening of the perturbed free electron gas; and this allows the GPT to go beyond the local density approximation by using a local field correction which amounts to treating the electron–electron interaction at the level of the random phase approximation. In our case, we use an energy dependent non local pseudopotential for Mg in AHS form [32] within a small core, screened self consistently to first order using the dielectric function of Ichimaru & Utsumi [33] based on the correlation energy of Vosko *et al*. [34]. The GPT for Mg in this form has been thoroughly tested against experiment and standard DFT calculations [12,23,24]. For example, the GPT predicts that the atomic volume is *Ω*_0_ = 160.9 bohr^3^ and the axial ratio is *c*/*a* = 1.621. In order to be comparing like with like, in our GPT calculations, we use the LMTO-DFT predicted lattice constants, see §§3a and 3bi.

#### (iii) Supercells

We build conventional supercells with the hcp *c*-axis normal to a rectangular area of basal plane. For Brillouin zone integrations in the LMTO calculations, we employ 60 divisions along the long axis of the Brillouin zone in the plane of the fault and 35 divisions along the short axis; there are six divisions along the reciprocal lattice vector normal to the basal plane and there are 12 (0002) planes separating the faults generated by the periodic boundary conditions. We use both modified tetrahedron integration [25], and smearing of the Fermi surface with a Fermi function at temperature equivalent to *kT* = 0.03 Ry: that is, an electron temperature of 4737 K. There is no Brillouin zone integration in the GPT; the total energy is simply given by summing equation (3.15) over all neighbours distant to *R*_*c*_ = 28.3 bohr. The oscillating function *v*_2_ is truncated smoothly to zero beyond *R*_*c*_ by replacing *v*_2_(*r*) in the range *R*_0_ < *r* < *R*_*c*_ by a degree five Hermite interpolating polynomial designed to match continuously and twice differentiably to *v*(*R*_0_) with *R*_0_ = 26.7 bohr and zero at *R*_*c*_.

## 4. Results

As we mention in §3b, a rather modest number of total energy calculations (table 2) yields a very large body of data when entered into the formulae of the AN^*N*^NI model. This is because *N* + 1 total energies furnish us with the AN^*N*^NI model parameters *J* up to *J*_*N*_ and these in turn lead to four stacking fault energies. The wealth of data extends beyond this since at any level of theory up to level five, we are free to choose which combinations of the seven total energies to use—that is to say, at level *N* we need to choose *N* + 1 total energies taken from the seven that we have available in table 2. This means that at level *N* we are free to make $\left(\begin{array}{c} 7\\ N+1 \end{array}\right)$ combinations giving that many approximations to each of the four stacking faults at that level. In fact, a particular difficulty is how to display the data in a form that can be apprehended by the reader, so that analysis can be made and conclusions reached. Before taking that route, we display in table 3 the results from just the first neighbour, Ising (‘ANNI’) model and their comparisons to our supercell benchmarks and some from the literature. We will return to this table in the discussion, §5, but it is worthwhile to point out here that there is a considerable scatter in the published SFEs despite them all being calculated using standard DFT. This acts as an indication that such calculations are very demanding and require complete control over convergence with respect to supercell length, basis set and **k**-point mesh. To begin the presentation of the full data, we have assembled into table 4, in the appendix, a unique designation number, *c*, for each combination of polytypes that may be used to yield a unique estimate of each of the four-fault energies. For the case of *N* = 1, any *two* of the polytypes may be chosen to provide 21 estimates of *J*_1_ in the Ising model. In the case *N* = 2, there are 35 ways of choosing three out of the seven polytypes, providing 35 estimates of *J*_1_ and *J*_2_. So in total, there are 21, 35, 35, 21 and 7 combinations for N=1−5. However, some of these turn out not to give solutions when equations (3.2)–(3.8) are inverted due to over completeness. Hence, there are fewer than the total possible number of combinations in table 4, leaving a total of 94 rows of data. In table 4, we also list the values of the *J*-parameters and the four fault energies deduced by inversion of the equations (3.2)–(3.8), the application of the GPT total energies from table 2 inserted into equations (3.11)–(3.14).

**Table 3.** Stacking fault energies calculated via the first neighbour Ising (ANNI) model and their standard deviations averaged over the 21 combinations for *N* = 1 (table 4), compared to the supercell results (S.c.), for each method used for the calculation of the total energy. The last column lists some fault energies calculated using DFT and taken from the literature.

	GPT		DFT (tetrahedron)		DFT (*kT* = 0.03 Ry)		
*γ* (mJ m^−2^)	ANNI av.	S.c.	ANNI av.	S.c.	ANNI av.	S.c.	literature
I_1_	19.4±2.0	17	24.8±6.9	19	25.2±3.1	28	21^a^, 18^b^, 20^c^, 14^d^, 16^e^, 21^f^, 18^g^, 17^i^
I_2_	38.7±4.0	34	49.7±13.9	35	50.3±6.2	48	44^a^, 36^b^, 48^d^,34^e^, 34^g^, 34^h^, 34^i^, 37^j^, 32^k^
E	58.1±6.0	52	74.5±20.8	51	75.5±9.3	66	69^a^, 58^b^, 99^d^, 59^e^, 54^g^
T	38.7±4.0	36	49.7±13.9	41	50.3±6.2	55	51^a^, 40^b^, 38^e^

^a^[15], ^b^[35], ^c^[18], ^d^[36], ^e^[37], ^f^[38], ^g^[29], ^h^[39], ^i^[40], ^j^[41], ^k^[42].

**Table 4.** All combinations, *c*, of polytypes are defined. Here, *N* refers to the number of neighbours included, so that the AN^*N*^NI model is used. As there is a total of 94 combinations, a number between 1 and 94 is assigned to each combination of polytypes. Moreover, interaction parameters *J*_*n*_ are displayed for each combination of structures, in units of meV, as well as stacking fault energies *γ* in units of mJ m^−2^. The energies shown are the results from GPT calculations.

*N*	*c*	2H	3R	4H	6H	9R	10H	15R	*J*_1_	*J*_2_	*J*_3_	*J*_4_	*J*_5_	*J*_6_	$\gamma_{{\text{I}}_{1}}$	$\gamma_{{\text{I}}_{2}}$	*γ*_E_	*γ*_T_
1	1	×	×						−5.4	—	—	—	—	—	19.3	38.5	57.8	38.5
	2		×	×					−5.3	—	—	—	—	—	19.2	38.3	57.5	38.2
	3		×		×				−5.7	—	—	—	—	—	20.4	40.8	61.1	40.8
	4		×				×		−5.9	—	—	—	—	—	21.2	42.4	63.7	42.4
	5		×			×			−5.8	—	—	—	—	—	20.6	41.3	61.9	41.3
	6		×					×	−5.5	—	—	—	—	—	19.8	39.5	59.3	39.5
	7	×		×					−5.4	—	—	—	—	—	19.4	38.8	58.2	38.8
	8	×			×				−5.2	—	—	—	—	—	18.7	37.4	56.2	37.4
	9	×					×		−5.2	—	—	—	—	—	18.8	37.6	56.4	37.6
	10	×				×			−4.6	—	—	—	—	—	16.5	33.1	49.6	33.1
	11	×						×	−5.3	—	—	—	—	—	18.9	37.9	56.8	37.9
	12			×	×				−4.7	—	—	—	—	—	16.7	33.4	50.1	33.4
	13			×			×		−5.0	—	—	—	—	—	17.8	35.6	53.3	35.6
	14			×		×			−7.0	—	—	—	—	—	25.1	50.3	75.4	50.3
	15			×				×	−4.7	—	—	—	—	—	16.7	33.4	50.1	33.4
	16				×		×		−5.3	—	—	—	—	—	19.1	38.2	57.3	38.2
	17				×	×			−5.8	—	—	—	—	—	20.9	41.8	62.8	41.8
	18				×			×	−4.7	—	—	—	—	—	16.7	33.4	50.1	33.4
	19					×	×		−5.7	—	—	—	—	—	20.4	40.8	61.2	40.8
	20						×	×	−5.1	—	—	—	—	—	18.3	36.6	54.9	36.6
	21					×		×	−6.1	—	—	—	—	—	22.0	43.9	65.9	43.9
2	22	×	×	×					−5.4	0.02	—	—	—	—	19.4	38.7	57.9	38.8
	23	×	×		×				−5.4	−0.2	—	—	—	—	18.2	37.4	56.7	36.3
	24	×	×				×		−5.4	−0.3	—	—	—	—	17.3	36.6	55.9	34.7
	25	×	×			×			−5.4	−0.4	—	—	—	—	16.5	35.8	55.1	33.1
	26	×	×					×	−5.4	−0.1	—	—	—	—	18.8	38.1	57.3	37.6
	27		×	×		×			−6.2	0.4	—	—	—	—	26.1	47.3	69.4	50.3
	28		×		×	×			−5.8	0.1	—	—	—	—	21.5	42.4	63.3	42.9
	29		×			×	×		−5.6	−0.2	—	—	—	—	18.9	39.0	59.1	37.8
	30		×			×		×	−6.0	0.3	—	—	—	—	23.3	44.8	66.4	46.6
	31	×		×	×				−5.0	0.2	—	—	—	—	19.4	37.4	55.5	38.8
	32	×		×			×		−5.2	0.1	—	—	—	—	19.4	38.0	56.6	38.8
	33	×		×				×	−5.0	0.2	—	—	—	—	19.4	37.4	55.5	38.8
	34	×			×		×		−5.3	−0.04	—	—	—	—	18.6	37.4	56.3	37.2
	35	×			×	×			−5.8	−0.6	—	—	—	—	16.5	37.4	58.4	33.1
	36	×			×			×	−5.0	0.2	—	—	—	—	19.4	37.4	55.5	38.8
	37	×				×	×		−5.4	−0.4	—	—	—	—	16.5	36.1	55.6	33.1
	38	×					×	×	−5.2	0.1	—	—	—	—	19.1	37.8	56.5	38.1
	39	×				×		×	−6.6	−1.0	—	—	—	—	16.5	40.3	64.1	33.1
	40			×	×	×			−5.8	0.6	—	—	—	—	25.1	46.0	67.0	50.3
	41			×		×	×		−6.0	0.5	—	—	—	—	25.1	46.6	68.0	50.3
	42			×		×		×	−5.8	0.6	—	—	—	—	25.1	46.0	67.0	50.3
	43				×	×	×		−5.8	0.3	—	—	—	—	22.7	43.6	64.6	45.5
	44				×	×		×	−5.8	0.6	—	—	—	—	25.1	46.0	67.0	50.3
	45					×	×	×	−5.9	0.5	—	—	—	—	24.2	45.4	66.6	48.3
3	46	×	×	×	×				−5.2	0.02	−0.2	—	—	—	20.6	38.7	57.9	40.0
	47	×	×	×			×		−5.1	0.02	−0.3	—	—	—	21.5	38.7	57.9	40.9
	48	×	×	×		×			−5.8	0.02	0.4	—	—	—	16.5	38.7	57.9	35.9
	49	×	×	×				×	−5.2	0.02	−0.2	—	—	—	20.6	38.7	57.9	40.0
	50	×	×		×	×			−5.5	−0.3	0.1	—	—	—	16.5	36.6	55.9	33.9
	51	×	×		×			×	−5.2	0.02	−0.2	—	—	—	20.6	38.7	57.9	40.0
	52	×	×			×	×		−5.4	−0.3	0.1	—	—	—	16.5	36.2	55.5	33.5
	53	×	×				×	×	−5.0	0.1	−0.4	—	—	—	23.1	39.5	58.8	43.4
	54	×	×			×		×	−5.6	−0.2	0.2	—	—	—	16.5	37.3	56.6	34.6
	55		×	×	×	×			−6.3	0.6	−0.2	—	—	—	28.8	50.9	74.3	56.4
	56		×	×		×	×		−6.5	0.7	−0.3	—	—	—	31.3	53.5	77.7	60.6
	57		×	×		×		×	−6.3	0.6	−0.2	—	—	—	28.8	50.9	74.3	56.4
	58		×		×	×		×	−6.3	0.6	−0.2	—	—	—	28.8	50.9	74.3	56.4
	59		×			×	×	×	−6.8	1.1	−0.4	—	—	—	36.4	59.4	85.3	69.9
	60	×		×	×		×		−5.1	0.1	−0.1	—	—	—	20.2	38.2	57.1	39.6
	61	×		×	×	×			−4.6	0.6	0.4	—	—	—	16.5	34.6	49.8	35.9
	62	×		×		×	×		−5.3	0.2	0.4	—	—	—	16.5	37.0	54.7	35.9
	63	×		×			×	×	−5.1	0.1	−0.1	—	—	—	20.2	38.2	57.1	39.6
	64	×		×		×		×	−4.6	0.6	0.4	—	—	—	16.5	34.6	49.8	35.9
	65	×			×	×	×		−5.4	−0.2	0.1	—	—	—	16.5	36.4	55.3	34.1
	66	×			×		×	×	−5.1	0.1	−0.1	—	—	—	20.2	38.2	57.1	39.6
	67	×			×	×		×	−4.6	0.6	0.4	—	—	—	16.5	34.6	49.8	35.9
	68	×				×	×	×	−5.4	0.0	0.3	—	—	—	16.5	36.7	55.0	34.9
	69			×	×	×	×		−6.2	0.6	−0.1	—	—	—	27.5	49.2	71.8	54.2
	70			×		×	×	×	−6.2	0.6	−0.1	—	—	—	27.5	49.2	71.8	54.2
	71				×	×	×	×	−6.2	0.6	−0.1	—	—	—	27.5	49.2	71.8	54.2
4	72	×	×	×	×		×		−5.3	0.02	−0.1	−0.1	—	—	18.1	37.8	56.3	37.5
	73	×	×	×	×	×			−5.5	0.02	0.1	−0.3	—	—	14.5	36.6	53.9	33.9
	74	×	×	×		×	×		−5.5	0.02	0.2	−0.2	—	—	14.9	37.0	54.7	34.3
	75	×	×	×			×	×	−5.3	0.02	−0.1	−0.1	—	—	18.1	37.8	56.3	37.5
	76	×	×	×		×		×	−5.5	0.02	0.1	−0.3	—	—	14.5	36.6	53.9	33.9
	77	×	×		×	×	×		−5.5	−0.1	0.1	−0.1	—	—	15.7	36.6	55.1	33.9
	78	×	×		×		×	×	−5.3	0.02	−0.1	−0.1	—	—	18.1	37.8	56.3	37.5
	79	×	×		×	×		×	−5.5	0.02	0.1	−0.3	—	—	14.5	36.6	53.9	33.9
	80	×	×			×	×	×	−5.5	−0.1	0.1	−0.2	—	—	15.2	36.9	54.8	34.1
	81		×	×	×	×	×		−6.0	0.4	−0.1	−0.1	—	—	22.9	45.0	65.8	47.1
	82		×	×		×	×	×	−6.0	0.4	−0.1	−0.1	—	—	22.9	45.1	65.8	47.1
	83		×		×	×	×	×	−6.0	0.4	−0.1	−0.1	—	—	22.9	45.1	65.8	47.1
	84	×		×	×	×	×		−5.7	−0.1	0.1	−0.3	—	—	14.1	37.0	54.7	33.5
	85	×		×		×	×	×	−5.7	−0.1	0.1	−0.3	—	—	14.1	37.0	54.7	33.5
	86	×			×	×	×	×	−5.7	−0.1	0.1	−0.3	—	—	14.1	37.0	54.7	33.5
5	87	×	×	×	×	×	×		−5.6	0.02	0.1	−0.3	0.1	—	13.3	36.6	53.3	33.3
	88	×	×	×	×		×	×	−5.3	0.02	−0.1	−0.1	0.0	—	18.1	37.8	56.3	37.5
	89	×	×	×	×	×		×	−5.5	0.02	0.1	−0.3	0.0	—	14.5	36.6	53.9	33.9
	90	×	×	×		×	×	×	−5.6	0.02	0.1	−0.3	0.05	—	14.0	36.8	53.9	33.7
	91	×	×		×	×	×	×	−5.7	0.2	0.1	−0.5	0.2	—	10.9	36.6	51.5	32.7
	92		×	×	×	×	×	×	−6.0	0.4	−0.1	−0.1	0.0	—	22.9	45.0	65.8	47.1
	93	×		×	×	×	×	×	−5.7	−0.1	0.1	−0.3	0.0	—	14.1	37.0	54.7	33.5
6	94	×	×	×	×	×	×	×	−5.5	−0.04	0.1	−0.2	0.0	0.1	16.1	37.0	55.5	34.7

**Figure 2.**
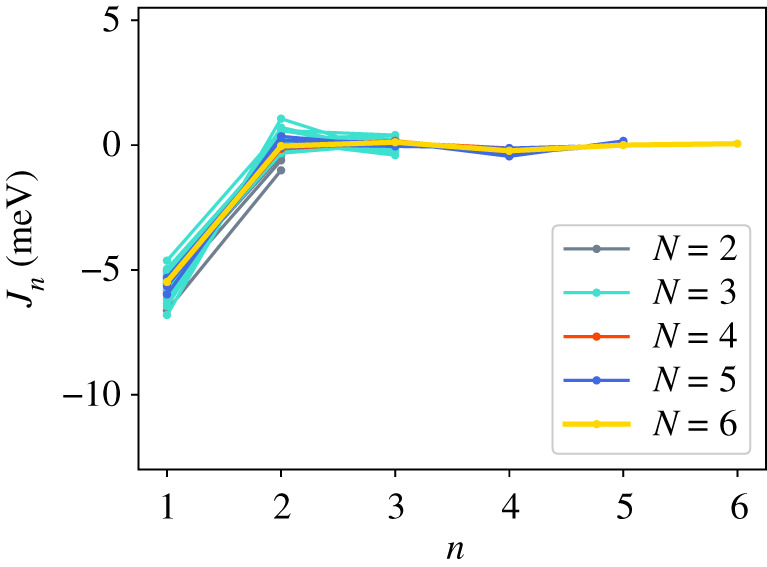
AN^*N*^NI model *J*-parameters, *J*_*n*_ calculated using GPT. (Online version in colour.)

**Figure 3.**
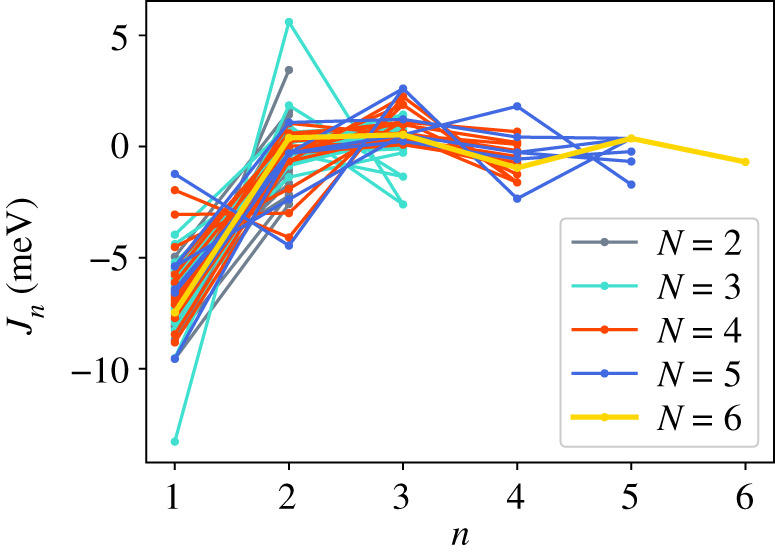
AN^*N*^NI model *J*-parameters, *J*_*n*_ calculated using LMTO-DFT with tetrahedron integration. (Online version in colour.)

**Figure 4.**
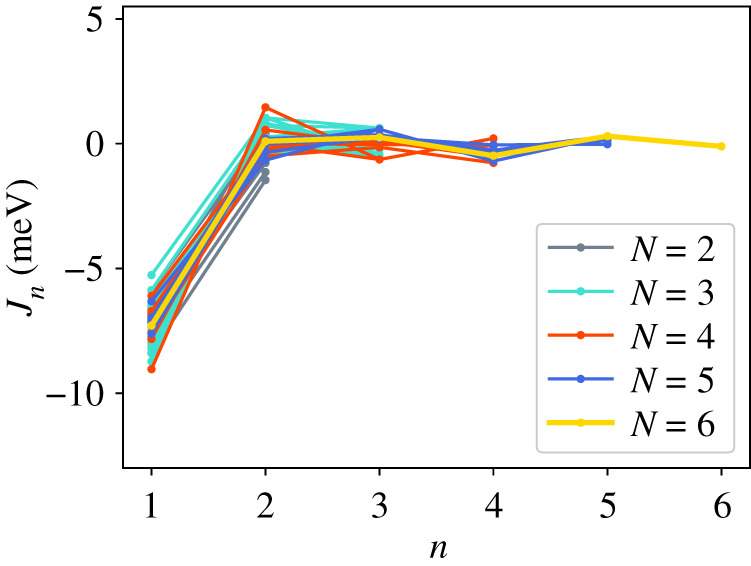
AN^*N*^NI model *J*-parameters, *J*_*n*_ calculated using LMTO-DFT with Fermi function smearing at *kT* = 0.03 Ry. (Online version in colour.)

In addition to the GPT total energies, we have two total energies from the LMTO-DFT calculations. In figures 2–4, we show the AN^*N*^NI model *J*_*n*_ parameters as functions of *n* plotted for all of our combinations from table 4. For the GPT total energies, the *J*-parameters for a particular *n* cluster quite closely together, while for the LMTO-DFT using the modified tetrahedron method there is considerable scatter. We find that this scatter can be largely removed at the cost of some precision by replacing the tetrahedon method in the Brillouin zone integration with a smearing of the Fermi cut-off with a Fermi function at temperature equivalent to *kT* = 0.03 Ry. We also observe that the *J*-parameters calculated using LMTO-DFT (figures 3–4) do not converge as rapidly towards zero as do the *J*-parameters calculated using GPT (figure 2). We will return to these observations in §5b. It is striking that the very evident contrast in the *J*-parameters using tetrahedron Brillouin zone integration or an electron temperature is concealed in the very small differences in polytype energies displayed in rows 2 and 3 in table 2. Although there seem to be very minor deviations in the energy differences in table 2 these clearly conspire to have dramatic consequences when equations (3.2)–(3.8) are inverted.

**Figure 5.**
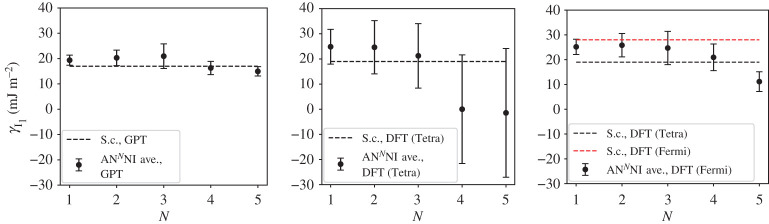
AN^*N*^NI model average $\gamma_{{\text{I}}_{1}}$ and standard deviation, calculated using the GPT, LMTO-DFT with tetrahedron integration and LMTO-DFT with electron temperature, *kT* = 0.03 Ry (labelled ‘Fermi’ in the legend). The dashed lines indicate the energy calculated using the supercell method for each total energy calculation method: $\gamma_{{\text{I}}_{1}}=17\;{\text{mJ m}}^{-2}$ (GPT), 19 mJ m^−2^ (DFT, tetrahedron), 28 mJ m^−2^ (DFT, *kT* = 0.03 Ry). (Online version in colour.)

**Figure 6.**
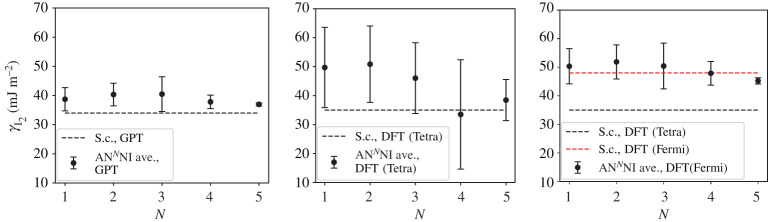
AN^*N*^NI model average $\gamma_{{\text{I}}_{2}}$ and standard deviation, calculated using the GPT, LMTO-DFT with tetrahedron integration and LMTO-DFT with electron temperature, *kT* = 0.03 Ry (labeled ‘Fermi’ in the legend). The dashed lines indicate the energy calculated using the supercell method for each total energy calculation method: $\gamma_{{\text{I}}_{2}}=34\;{\mathrm{mJm}}^{-2}$ (GPT), 35 mJ m^−2^ (DFT, tetrahedron), 48 mJ m^−2^ (DFT, *kT* = 0.03 Ry). (Online version in colour.)

**Figure 7.**
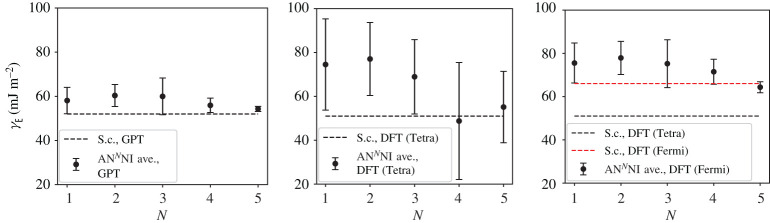
AN^*N*^NI model average *γ*_E_ and standard deviation, calculated using the GPT, LMTO-DFT with tetrahedron integration and LMTO-DFT with electron temperature, *kT* = 0.03 Ry (labelled ‘Fermi’ in the legend). The dashed lines indicate the energy calculated using the supercell method for each total energy calculation method: *γ*_E_ = 52 mJ m^−2^ (GPT), 51 mJ m^−2^ (DFT, tetrahedron), 66 mJ m^−2^ (DFT, *kT* = 0.03 Ry). (Online version in colour.)

**Figure 8.**
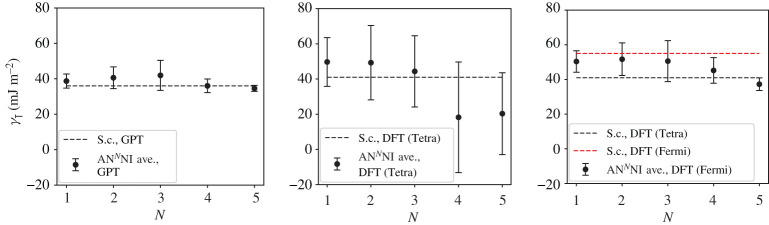
AN^*N*^NI model average *γ*_T_ and standard deviation, calculated using the GPT, LMTO-DFT with tetrahedron integration and LMTO-DFT with electron temperature, *kT* = 0.03 Ry (labelled ‘Fermi’ in the legend). The dashed lines indicate the energy calculated using the supercell method for each total energy calculation method: *γ*_T_ = 36 mJ m^−2^ (GPT), 41 mJ m^−2^ (DFT, tetrahedron), 55 mJ m^−2^ (DFT, *kT* = 0.03 Ry). (Online version in colour.)

**Figure 9.**
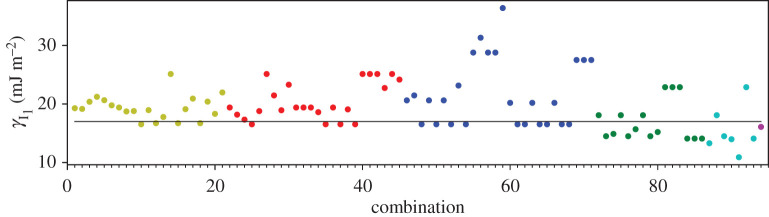
I_1_ stacking fault energy for each of the 94 combinations in table 4. Different colours indicate different *N*—the maximum number of neighbours taken into account—with *N* + 1 structures in each combination. The solid black line indicates the energy calculated using the supercell method, $\gamma_{{\text{I}}_{1}}=17\;{\mathrm{mJm}}^{-2}$. Calculations used the GPT. (Online version in colour.)

**Figure 10.**
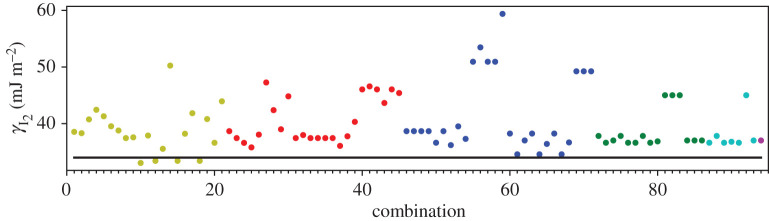
I_2_ stacking fault energy for each of the 94 combinations in table 4. Different colours indicate different *N*—the maximum number of neighbours taken into account—with *N* + 1 structures in each combination. The solid black line indicates the energy calculated using the supercell method, $\gamma_{{\text{I}}_{2}}=34\;{\mathrm{mJm}}^{-2}$. Calculations used the GPT. (Online version in colour.)

**Figure 11.**
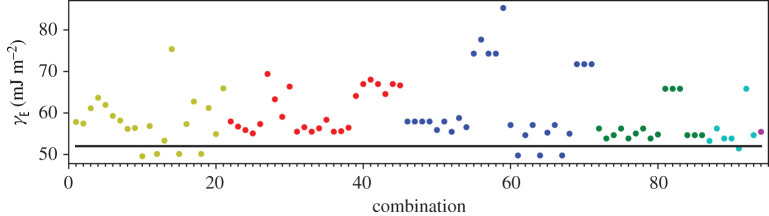
Extrinsic stacking fault energy for each of the 94 combinations in table 4. Different colours indicate different *N*—the maximum number of neighbours taken into account—with *N* + 1 structures in each combination. The solid black line indicates the energy calculated using the supercell method, *γ*_E_ = 52 mJ m^−2^. Calculations used the GPT. (Online version in colour.)

**Figure 12.**
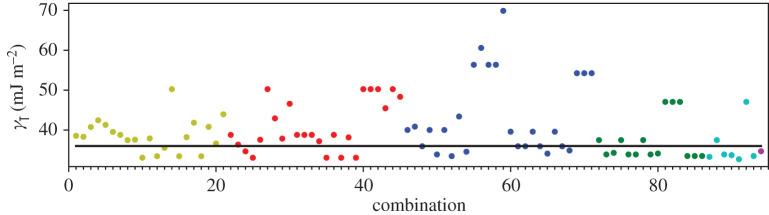
Twin-like fault energy for each of the 94 combinations in table 4. Different colours indicate different *N*—the maximum number of neighbours taken into account—with *N* + 1 structures in each combination. The solid black line indicates the energy calculated using the supercell method,*γ*_T_ = 36 mJ m^−2^. Calculations used the GPT. (Online version in colour.)

**Figure 13.**
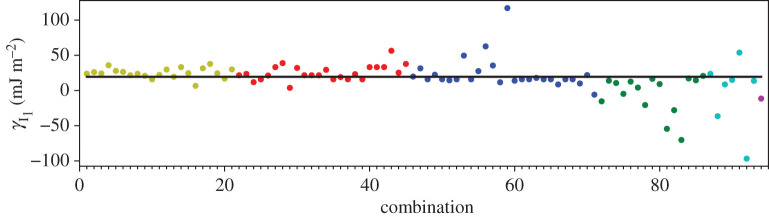
I_1_ stacking fault energy for each of the 94 combinations in table 4. Different colours indicate different *N*—the maximum number of neighbours taken into account—with *N* + 1 structures in each combination. The solid black line indicates the energy calculated using the supercell method, $\gamma_{{\text{I}}_{1}}=19\;{\mathrm{mJm}}^{-2}$. Calculations used LMTO-DFT and tetrahedron integration. (Online version in colour.)

**Figure 14.**
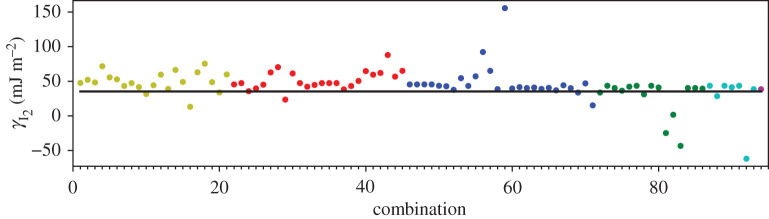
I_2_ stacking fault energy for each of the 94 combinations in table 4. Different colours indicate different *N*—the maximum number of neighbours taken into account—with *N* + 1 structures in each combination. The solid black line indicates the energy calculated using the supercell method, $\gamma_{{\text{I}}_{2}}=35\;{\text{mJ m}}^{-2}$. Calculations used LMTO-DFT and tetrahedron integration. (Online version in colour.)

**Figure 15.**
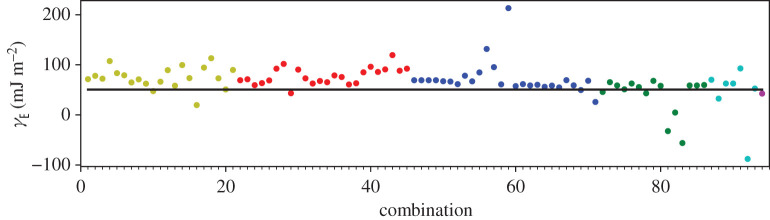
Extrinsic stacking fault energy for each of the 94 combinations in table 4. Different colours indicate different *N*—the maximum number of neighbours taken into account—with *N* + 1 structures in each combination. The solid black line indicates the energy calculated using the supercell method, *γ*_E_ = 51 mJ m^−2^. Calculations used LMTO-DFT and tetrahedron integration. (Online version in colour.)

**Figure 16.**
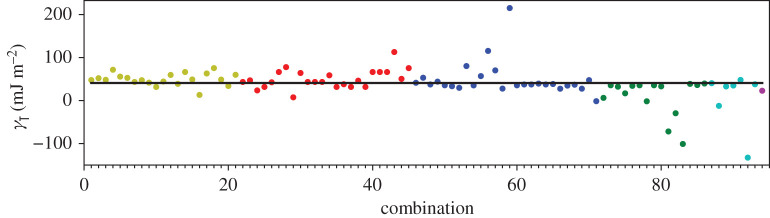
Twin-like fault energy for each of the 94 combinations in table 4. Different colours indicate different *N*—the maximum number of neighbours taken into account—with *N* + 1 structures in each combination. The solid black line indicates the energy calculated using the supercell method, *γ*_T_ = 41 mJ m^−2^. Calculations used LMTO-DFT and tetrahedron integration. (Online version in colour.)

**Figure 17.**
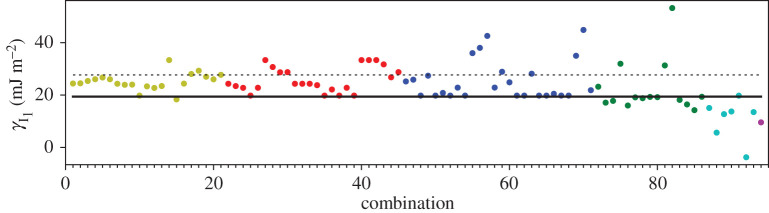
I_1_ stacking fault energy for each of the 94 combinations in table 4. Different colours indicate different *N*—the maximum number of neighbours taken into account—with *N* + 1 structures in each combination. The dotted black line indicates the energy calculated using the supercell method, $\gamma_{{\text{I}}_{1}}=28\;{\text{mJ m}}^{-2}$. Calculations used LMTO-DFT and an electron temperature, *kT* = 0.03 Ry. The solid black line indicates the energy calculated using the supercell method, $\gamma_{{\text{I}}_{1}}=19\;{\text{mJ m}}^{-2}$, calculated using LMTO-DFT and tetrahedron integration. (Online version in colour.)

**Figure 18.**
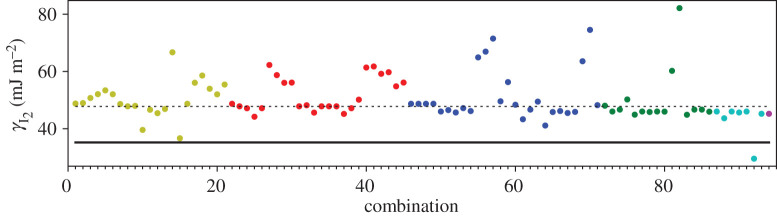
I_2_ stacking fault energy for each of the 94 combinations in table 4. Different colours indicate different *N*—the maximum number of neighbours taken into account—with *N* + 1 structures in each combination. The dotted black line indicates the energy calculated using the supercell method, $\gamma_{{\text{I}}_{2}}=48\;{\text{mJ m}}^{-2}$. Calculations used LMTO-DFT and an electron temperature, *kT* = 0.03 Ry. The solid black line indicates the energy calculated using the supercell method, $\gamma_{{\text{I}}_{2}}=35\;{\text{mJ m}}^{-2}$, calculated using LMTO-DFT and tetrahedron integration. (Online version in colour.)

**Figure 19.**
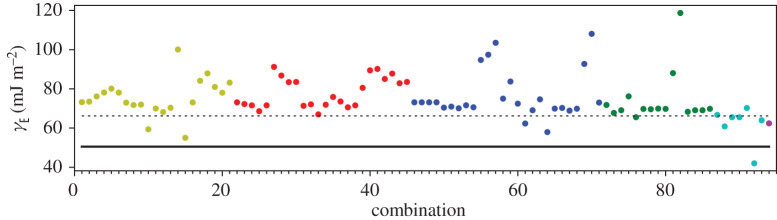
Extrinsic stacking fault energy for each of the 94 combinations in table 4. Different colours indicate different *N*—the maximum number of neighbours taken into account—with *N* + 1 structures in each combination. The dotted black line indicates the energy calculated using the supercell method,*γ*_E_ = 66 mJ m^−2^. Calculations used LMTO-DFT and an electron temperature, *kT* = 0.03 Ry. The solid black line indicates the energy calculated using the supercell method, *γ*_E_ = 51 mJ m^−2^, calculated using LMTO-DFT and tetrahedron integration. (Online version in colour.)

**Figure 20.**
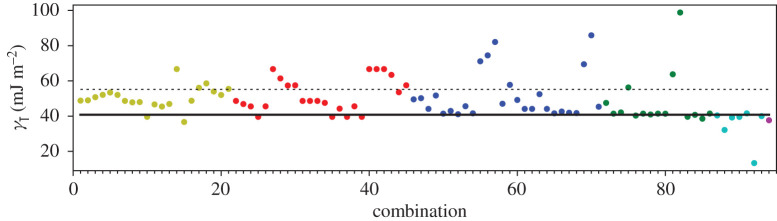
Twin-like fault energy for each of the 94 combinations in table 4. Different colours indicate different *N*—the maximum number of neighbours taken into account—with *N* + 1 structures in each combination. The dotted black line indicates the energy calculated using the supercell method, *γ*_T_ = 55 mJ m^−2^. Calculations used LMTO-DFT and an electron temperature, *kT* = 0.03 Ry. The solid black line indicates the energy calculated using the supercell method, *γ*_T_ = 41 mJ m^−2^, calculated using LMTO-DFT and tetrahedron integration. (Online version in colour.)

A third way to display the data is presented in figures 9–20 in the Appendix. Here, we show as ordinates each estimate of each fault energy as calculated from each of the 94 combinations from table 4 as abscissa to each plot. It will be possible for the reader to identify each calculation and in particular its excursion from the benchmark supercell SFE with one of the 94 combinations. In each of figures 9–20, we show as horizontal lines fault energies calculated using the supercell method described in [42]. There is evidently considerable scatter in these data and convergence is not convincingly demonstrated. This is to be expected in a free electron metal as will be discussed in §5. On the other hand much of the scatter, which is also evident in figures 2–4 can be traced to certain ‘outliers’ in figures 9–20. As we explain in the appendix, we can eliminate much of the noise by excluding results from those combinations in table 4 which include all of the three longest period polytypes, 9R, 10H and 15R. We then plot the predicted SFEs for each fault and for each of the three total energy methods in figures 5–8. We defer discussion of these results to §5c.

## 5. Discussion

### (a) Benefits of the AN^*N*^NI model, and the Ising model first neighbour approximation

As far as we are aware, there are few or even no direct measurements of stacking fault energies in Mg. Normally only the intrinsic fault energy can be measured and we are aware of one experimental measure that places the energy of the I_2_ fault at lower than 50 mJ m^−2^ [43]. This argues strongly for the need of theory to approach the question. Traditionally fault energies have been calculated using the supercell method in DFT and whereas this was expensive in the past, leading to the application of the Ising and ANNNI models to the problem, with current computers it is not particularly challenging to calculate fault energies (notwithstanding the considerable uncertainty in the literature—table 3). On the other hand, the approach we take here has a number of advantages.

1. Clear insight is obtained into the relation between structural energy differences between polytypes and stacking fault and twin boundary energies.
2. The method is in principle extensible to alloys, for which the supercell method becomes cumbersome. In addition, there is the promise of much greater insight since the theory of alloy stability and the effects of impurities on the electronic structure of metals may be transposed into an understanding of the role of alloying and impurities on fault energies and through that understanding to the plastic response of an engineering alloy. This is a matter for further work.
3. A possible disadvantage is that whereas using the AN^*N*^NI model does not require us to work with ideal axial ratio hcp metals, any relaxation of the interplanar spacing or atomic positions on creating the defect are neglected. In the present case, however, these are not limitations. The intrinsic stacking fault energy calculated with the supercell method and neglecting relaxation is 35 mJ m^−2^ (table 3), whereas using the same DFT program and lattice constants but allowing relaxation the result is 32.1 mJ m^−2^ [42].
4. As an example of insight gained, it is clear from equations (3.11) to (3.14) that at the level of nearest neighbours we have the relation,
  5.1 $$\gamma_{{\text{I}}_{1}}=\frac{1}{2}\gamma_{{\text{I}}_{2}}=\frac{1}{3}\gamma_{\text{E}},$$
  and indeed this is reasonably well adhered to in the supercell calculations in table 3, at least in respect of I_1_ and I_2_. We also have at the Ising (ANNI) level that $\gamma_{{\text{I}}_{2}}=\gamma_{\text{T}}$. These relations expose the well known first approximation to the fault energies as proportional to energy differences between fcc and hcp crystals [3–5,18].

### (b) Convergence of the *J*-parameters

We turn next to a discussion of the series (3.1) taken beyond second neighbours. It is rather clear from table 3 that the first neighbour Ising model leads to a faithful rendering of the four fault energies compared to supercell calculations, particularly if the GPT is employed in the total energy calculation. However, the ANNI relation $\gamma_{{\text{I}}_{2}}=\gamma_{\text{T}}$ is not supported by the supercell results. This is to be anticipated in view of the long range of atomic interactions expected in a free electron metal (figure 1), and it is important to examine the convergence of further neighbour interactions. We are not aware from the literature of any study which makes such a thorough examination of the convergence of what we call the AN^*N*^NI model as we have done here. First, we have examined the convergence of the *J*-parameters; and second, we have considered every possible combination of up to seven polytypes in order to check the method for internal consistency. Our full sets of data are presented in the appendix.

Whereas figures 2–4 and table 4 show that the *J*_*n*_ for *n* > 1 are much smaller in magnitude than *J*_1_, the subsequent convergence is rather slow, as is to be expected in a free electron metal. One reason for this is that oscillations in the *J*_*n*_ as functions of *n* are not sufficiently damped (table 4), especially in the case of the LMTO with tetrahedron integration; if *J*_*N*_ has not approached zero at the point that we truncate the AN^*N*^NI model then neglect of the unknown slowly decaying *J*_*n*_, *n* > *N* will lead to the inconsistency across different combinations and scatter in the SFEs that we observe. A second reason is outside the control of the precision in total energy calculations and arises from the fact that the *prefactors* to the *J*-parameters do not get smaller with each order of approximation, both in the expression for the polytype energies (3.2)–(3.8) and for the fault energies (3.11)–(3.14). On the other hand, there is a very clearly increased scatter in figure 3 in the case of the LMTO-DFT with tetrahedron integration compared to the same parameters calculated using the GPT (figure 2). This scatter is removed in the LMTO-DFT if we replace the most precise Brillouin zone integration with the approximation of applying an electron temperature (figure 4). What the latter does, most significantly, is to change the abrupt discontinuity of occupancy of the one-electron eigenstates into a smooth transition. This is exactly equivalent to removing the logarithmic singularity in the energy–wavenumber characteristic in the theory of simple metals. The question then arises, whether and how this is evidently achieved in the GPT. One answer is that we implicitly smooth the Fermi surface discontinuity by applying a cut-off to the pair potential. This is indicated in figure 1 as two vertical lines: the pair potential is not taken to an infinite number of neighbours in the computation but is smoothly interpolated to zero using a polynomial which replaces *v*_2_ between two distances which we choose to be 26.7 bohr and 28.3 bohr [24]. While applying an appropriate electron temperature in the LMTO-DFT has the desirable effect of removing the excessive scatter in the *J*-parameters there is a price to pay, namely a loss of overall precision in the solution to the Kohn–Sham equations. This is reflected in table 3 which shows that the supercell fault energies using the finite electron temperature are not as well in accord with the most precise LMTO-DFT values which in turn are in very good agreement with the GPT. The conclusion must be that the GPT is not only as accurate as the standard DFT in predicting properties of Mg, it manages to deal with the screening of the free electron gas better than the standard DFT for which either at zero temperature there is scatter in the *J*_*n*_ or at finite temperature there is a loss of precision. We cannot be absolutely certain from where this benefit of the GPT over the DFT bandstructure method arises; however, a comparison with the analytical pair potential of Pettifor and Ward is instructive [44]. These authors fitted a *rational* approximation to the Lindhard function so that their derived energy–wavenumber characteristic had no singularity at twice the Fermi wavevector. This effectively damps the pair potential, but unlike our *v*_2_ which is only damped beyond *R*_0_, the Pettifor–Ward pair potential is damped at all *r*: the peaks are lowered and the troughs are filled in [30]. This circumstance, in our opinion, corresponds to the case of temperature smearing of the discontinuity in occupation number in our LMTO-DFT calculations: in that case all states in *reciprocal* space that are within *kT* of the Fermi surface are affected by the electron temperature, just as all interactions in *real* space are affected by the Pettifor–Ward sextic interpolation replacing the Lindhard screening function.

### (c) Convergence of the fault energies

As long as we sensibly remove outliers (see appendix) then we clearly see the convergence of the *J*-parameters, as discussed in §5b, reflected in the mean and standard deviation of the stacking fault energies. Figures 5–8 show the SFEs calculated using GPT as functions of *N* in the AN^*N*^NI model. Convergence is rather clearly demonstrated and there is a useful, if not dramatic, benefit of increasing *N*, when compared to the supercell result. The diametrically opposite situation is found for the LMTO-DFT using tetrahedron integration. Our conclusions of §5b concerning the weak damping of the oscillations in *J*_*n*_, shown in figure 3, are confirmed in the large standard deviations and indeed *negative* predicted mean SFEs in the cases of the growth, I_2_, and twin-like faults. As expected in view of our discussion in §5b these issues are largely, but not fully, resolved with the use of a large electron temperature. The plots of SFEs calculated using LMTO-DFT and the electron temperature in figures 5–8 now show by and large a narrowing of the error bars as *N* is increased, and in the cases of the deformation and extrinsic faults significant convergence to the supercell result. But it must be recalled that the supercell result itself suffers from the error in Brillouin zone integration compared to the tetrahedron method.

It is important to point out that in the cases of the GPT and LMTO-DFT with tetrahedron integration the outcome of the *N* = 6 AN^*N*^NI model is extremely close to the supercell result for all stacking fault energies, see figures 9–16. This argues strongly for the correctness of the theory.

## 6. Conclusion

We have been motivated to calculate to the best available precision the energies of the four basal stacking faults in the hexagonal close packed, free electron metal, magnesium. While these are critical control parameters in the development of microstructure and mechanical properties, they are almost totally inaccessible to experiment so that recourse to theory is mandated.

A thorough investigation was made of the precision and convergence of the ‘ AN^*N*^NI model’: an Ising model describing the total energy of crystals that may be described by a packing sequence of close-packed planes, extended to a range of inter-plane interactions up to the sixth neighbour. By a suitable limiting process, the parameters obtained from the study of polytypes may be used to obtain the energies of isolated stacking faults [3,5]. We have chosen a particularly challenging case, that of the free electron metal magnesium, whose interatomic forces are known to be of a particularly long range due the prominence of the discontinuity of electron occupancy across the nearly free electron Fermi surface. This results in a weak logarithmic singularity in the energy–wavenumber characteristic in *reciprocal* space: in turn leading to very weak damping of the screening of the pseudopotential (Friedel oscillation) in *real* space. These are well captured within the GPT by an analytic pair potential, *v*_2_, figure 1. Its range is particularly extended in the free electron metals, compared to the range of *v*_2_ in transition metals [45].

A feature of the AN^*N*^NI model is that a small number of high precision total energy calculations of *N* + 1 polytypes provides the first *N* *J*-parameters of the model. In addition, at each level of the expansion, we have a choice of up to $\left(\begin{array}{c} 7\\ N+1 \end{array}\right)$ combinations of polytype each in principle leading to equal values for the *J*-parameters. The deviation from this equality is a measure of the internal consistency of the model. This deviation was illustrated by points of the same colour in figures 9–20. These figures illustrated the cases of the three choices we made for finding the total energies of the polytypes, table 2, namely the GPT and the DFT as implemented in the full potential LMTO method [26]. We have validated our AN^*N*^NI model results against conventional ‘supercell’ calculations of fault energies. Our conclusions are as follows.

— Our benchmark calculations are the four fault energies calculated using supercells in the LMTO implementation of DFT [26] using modified tetrahedron method in the Brillouin zone integrations [25]—table 3, column 5. There is excellent agreement between these and our results using the GPT (column 3), indicating that the approximations to the DFT made in constructing the GPT pair potential, *v*_2_, are validated [12,24].
— As is evident from table 3, already at the level of first neighbours—the Ising model—fault energies are in reasonable agreement with supercell calculations using the same level of theory. In addition, relations between the fault energies within the ANNI model (5.1) are *reasonably well* adhered to in the supercell calculations (better using the GPT than the LMTO-DFT), although the extrinsic fault energy is significantly smaller in benchmark than predicted by equation (5.1). The Ising (ANNI) model prediction $\gamma_{{\text{I}}_{2}}=\gamma_{\text{T}}$ is not supported by the benchmark calculations. This means that corrections to the Ising model are desirable.
— If such corrections are to be made then as seen in the remaining data of this paper, care must be taken. In our most precise calculations, the LMTO-DFT with tetrahedron integration, we find that the AN^*N*^NI model expansion up to *N* = 6 results in *J*-parameters that are oscillating and insufficiently close to zero to regard the expansion (3.1) to be converged (figure 3). This is a principal conclusion of the this paper: the AN^*N*^NI model for a free electron metal *is slow to converge*. The symptom of this slow convergence is scatter in the values of the *J*-parameters obtained by using different choices of polytype combinations; scatter in the predicted fault energies, figures 13–16; and also in an error in the intrinsic fault energy in the Ising model, with large standard deviation as seen in column 4, table 3. We attribute this poor convergence to the sharp occupancy cut-off at the Fermi surface and confirm this supposition by making identical LMTO-DFT total energy calculations but with a finite electron temperature designed to smooth the zero temperature Heaviside step in the Fermi function. Convergence is thereby restored—figure 4. However, a heavy price is to pay in the consequent loss of precision as seen in column 7, table 3, which shows that such a large smearing in the Brillouin zone integrals leads to errors in the supercell results for the fault energies, when compared to the tetrahedron method.
— We find that the GPT offers good convergence of the *J*-parameters figure 2, with scatter in the fault energies consistent with the high electron temperature in LMTO-DFT, figures 9–12, *but without the attendant loss of precision* as is evident from the supercell results in table 3. This is a key conclusion of this paper. The damping of the logarithmic singularity in the energy–wavenumber characteristic is effected not in *reciprocal space* [44], but in *real space* [24] by a cut-off in the long-ranged part of the pair potential, figure 1; this does not compromise the precision of the total energy expression (3.15).

A matter for further work is the question of whether the inclusion of three-spin or even four or more spin terms in the AN^*N*^NI model may improve either precision or convergence. Our surmise is that it will not and that such an extension to the theory takes us beyond the simple context of the Ising description. Indeed, as we point out at the end of §5c, our longest ranged, *N* = 6, AN^*N*^NI model is in excellent agreement with our benchmark calculations, both the GPT and LMTO-DFT, in respect of all four stacking fault energies. Inclusion of three-spin terms would allow us to test the hypothesis that long-ranged interactions in the pairwise theory may be accounted for by first neighbour many-spin terms, and this remains a matter for future work.

## Data accessibility

All input files used for the calculation of the total energies of the polytypes of magnesium as well as supercells can be found at a public GitHub repository http://doi.org/10.5281/zenodo.4059479. The questaal suite, including the LMTO-DFT code, is available at https://www.questaal.org. The GPT code is available as part of the LAMMPS code at https://lammps.sandia.gov.

## Appendix A

Table 4 indicates all of the combinations of polytypes from table 1 that have been used to calculate the parameters of the AN^*N*^NI model, as displayed in figures 2–4. Not all the $\left(\begin{array}{c} 7\\ N+1 \end{array}\right)$ combinations at each level, *N*, of the AN^*N*^NI model can be used as the equations (3.2)–(3.8) may become overcomplete. Figures 9–20 show all of the calculated fault energies, with each set of a given level of approximation, *N*, in the AN^*N*^NI model indicated by a different colour. There is evident scatter in the data. Something might be learned by considering some of the obvious outliers. Notable cases are combinations 14, 21, 59, 83, 92. Combination 59 is clearly leading to error even in the normally well-behaved GPT. While this is not a hard and fast rule the tendency is for the outliers to be made up of combinations more largely taken from the longer period polytypes; and those whose interatomic forces are not dictated by symmetry to be zero, namely 6H and longer period polytypes. It is also notable that combinations that do not include both the ‘ferromagnetic’ and ‘antiferromagnetic’ polytypes, 3R and 2H, tend to provide less faithful estimates of the correct fault energies. These are useful guidelines for future applications of the AN^*N*^NI model and provide some scope for further investigation. For the present investigation, in order not to be swamped by the intrusion of outliers, we systematically remove from the averaging in figures 5–8 combinations that include all three of the longest period polytypes: 9R, 10H and 15R.
